# Duloxetine and Cognitive Behavioral Therapy with Phone-based Support for the Treatment of Chronic Musculoskeletal Pain: Study Protocol of the PRECICE Randomized Control Trial

**DOI:** 10.21203/rs.3.rs-3924330/v1

**Published:** 2024-04-15

**Authors:** Dennis C Ang, Swetha Davuluri, Sebastian Kaplan, Francis Keefe, Christine Rini, Christopher Miles, Haiying Chen

**Affiliations:** Wake Forest University School of Medicine; Wake Forest University School of Medicine; Wake Forest University School of Medicine; Duke University; Northwestern University; Wake Forest University School of Medicine; Wake Forest University

**Keywords:** Chronic musculoskeletal pain, duloxetine, cognitive behavioral therapy, motivational interviewing

## Abstract

**Background:**

Chronic musculoskeletal pain (CMP) is the most common, disabling, and costly of all pain conditions. While evidence exists for the efficacy of both duloxetine and web-based cognitive behavioral therapy (CBT) as monotherapy, there is a clear need to consider study of treatment components that may complement each other. In addition, given the reported association between patient’s adherence and treatment outcomes, strategies are needed to enhance participant’s motivation to adopt and maintain continued use of newly learned pain coping skills from CBT.

**Methods:**

Two hundred eighty participants will be recruited from the primary care clinics of a large academic health care system in North Carolina. Participants with CMP will be randomized to one of 3 treatment arms: (1) combination treatment (duloxetine + web-based self-guided CBT) with phone-based motivational interviewing (MI), (2) combination treatment without phone-based MI and (3) duloxetine monotherapy. Participants will be in the study for 24 weeks and will be assessed at baseline, week 13, and week 25. The primary outcome is the Brief Pain Inventory (BPI)-Global Pain Severity score, which combines BPI pain severity and BPI pain interference. Secondary measures include between-group comparisons in mean BPI pain severity and BPI pain interference scores. Data collection and outcome assessment will be blinded to treatment group assignment.

**Discussion:**

This randomized controlled trial (RCT) will determine if combination treatment with duloxetine and web-based CBT is superior to duloxetine monotherapy for the management of CMP. Furthermore, this RCT will determine the effectiveness of phone-based motivational interviewing in promoting the continued practice of pain coping skills; thereby, enhancing treatment outcomes.

**Trial Registration:**

NCT04395001. Registered in ClinicalTrials.gov on May 15, 2020.

## Introduction

### Background and rationale:

Pain is the most common symptom reported in primary care and accounts for over 100 million ambulatory encounters in the United States (US) each year.^1^ Pain costs the US over $550 billion each year in health care and lost productivity.^2^ Musculoskeletal pain is consistently the most common, disabling, and costly of all pain complaints.^1,3^ The functional and economic impact of musculoskeletal pain on both the working and the retired population is enormous.^4^ Chronic musculoskeletal pain (CMP) can have a profound negative impact on an individual’s physical, emotional and social well-being.^5–8^

Despite the enormous individual and societal burden of CMP, pain management remains suboptimal. Pharmacological treatments often provide minimal pain relief when used alone,^9^ and opioids for chronic non-musculoskeletal pain are fraught with problems. Despite a 30-fold increase in the global use of opioids over the past 30 years,^10^ opioids themselves produce only minimal reductions in chronic pain^9,11,12^ and may fail to improve or may even worsen functional status even when they do alleviate the pain.^13,14^

The underlying mechanisms driving musculoskeletal pain are not fully understood.^15^ Self-reported pain severity and dysfunction are poorly correlated with the extent of peripheral tissue abnormalities.^16,17^ This poor correlation has prompted a shift in research to focus on central pain processing abnormalities as the primary driver of chronic pain.^18,19^ Augmented central nervous system (CNS) processing of nociceptive signals and dysfunctional endogenous pain inhibition have been identified as characteristics of many musculoskeletal pain conditions including low back pain, osteoarthritis, and fibromyalgia.^20–24^

Augmented CNS processing of nociceptive signals and dysfunctional endogenous pain inhibition contribute to central sensitization. Central sensitization manifests as pain hypersensitivity, particularly tactile allodynia (painful response to a normally innocuous touch), pressure hyperalgesia, and enhanced temporal summation.^25^ In preclinical and clinical studies of pain, duloxetine, a selective serotonin norepinephrine reuptake inhibitor, reduces central sensitization.^26–29^ Based on the strength of efficacy data,^30^ duloxetine received a Food and Drug Administration (FDA) indication for CMP. However, when used in real world clinic practice, duloxetine only led to modest improvements in pain-related outcomes.^31,32^

Among the non-pharmacologic treatment approaches, cognitive behavioral therapy (CBT) has the strongest support from the literature. Substantial evidence exists for the benefits of CBT in CMP including low back pain, neck pain, temporo-mandibular joint pain, knee osteoarthritis, and fibromyalgia.^33^ CBT may also decrease central sensitization.^34^ Unfortunately, despite the proven efficacy of CBT, access to this behavioral pain management is a major limitation. To improve access, web-based CBT was developed.^35^ Systematic reviews have reported small but clinically significant improvements in several important domains including disability and pain severity.^36,37^ Nonetheless, the efficacy of web-based CBT has not been consistently observed partly due to variation in participants’ adherence on application of cognitive and behavioral pain coping skills.^36,37^ To improve adherence to web-based CBT skills for pain, motivational interviewing (MI) may be useful as it has been effective in counseling to elicit behavioral change.^38–41^

Overall, pharmacological or psychologically based monotherapy produces only modest reductions in pain.^42,43^ The multifaceted nature of CMP suggests that treatment programs that combine pharmacologic and nonpharmacologic therapies are essential to achieve the best outcomes.

### Objectives:

The primary objective of the Pain Response Evaluation of a Combined Intervention to Cope Effectively (PRECICE) study is to determine if combination treatment of duloxetine and web-based CBT with and without phone-based MI is superior to duloxetine monotherapy in improving pain-related outcomes. Concurrently, we seek to assess the effect of motivational interviewing to increase participants’ motivation to practice pain coping skills in enhancing treatment outcomes.

### Trial design:

The PRECICE study is a 24-week randomized controlled trial. Primary care patients with CMP will be randomized to one of three treatment arms: 1. combination treatment [duloxetine and web-based self-guided CBT] with phone-based MI, 2. combination treatment without phone-based MI, and 3. duloxetine therapy alone. The participants will be evaluated at baseline, week 13 (mid-point), and week 25 (last visit) of the study timeline (Figure 1).

## Methods

The methods for this study have been reported in accordance with the Standard Protocol Items: Recommendations for Interventional Trials (SPIRIT) reporting guidelines.^44^ [See Additional File 1]

### Setting:

The participants will be recruited from the primary care clinics of Atrium Health Wake Forest (WF) Baptist in North Carolina. There are 271 primary care providers who use the same electronic medical record (EMR) system (EPIC) from which patients are included. Atrium Health WF Baptist is in the urban area that also serves patients in the rural and suburban region of central North Carolina.

### Eligibility criteria:

The study inclusion criteria include the following: (1) Age ≥ 18 years; (2) daily pain for 3 months or longer affecting the low back, neck, hip, knee or widespread pain; and (3) at least moderate in BPI Global Pain Severity (GPS), defined as a GPS score of 5 or greater.^45,46^

Study exclusion criteria include (1) current use of duloxetine; (2) active suicidal ideation; (3) planned elective surgery during the study period to avoid the confounding effect of possible complicated post-surgery recovery course on the primary outcome; (4) ongoing unresolved disability claims; (5) comorbid conditions such as uncontrolled hypertension, inflammatory arthritis, cancer-related musculoskeletal pain, pregnancy, bipolar disorder or schizophrenia, narrow angle glaucoma, severe renal impairment (creatinine clearance <30); (6) current use of any of the following medications: tricyclic antidepressant > 25 mg daily dose, monoamine oxidase inhibitors, fluoxetine, sertraline, paroxetine, citalopram, escitalopram, venlafaxine, milnacipran, mirtazapine, gabapentin, aripiprazole, serotonin precursors (e.g., tryptophan), and strong CYP1A2 inhibitors (e.g., fluoroquinolones, fluvoxamine and verapamil); and (7) polypharmacy defined as concurrent daily use of 4 or more centrally acting medications such as anxiolytics, hypnotics, antipsychotics, and anticonvulsants (i.e., gabapentin and pregabalin).

### Informed consent:

Participants complete informed consent at the first in person visit (IPV). Consent forms will contain a detailed description of the study intervention, study procedures, and risks. Every attempt will be made to send the informed consent through regular mail or email to participants before the first study visit to allow participants to carefully review the informed consent document.

#### Interventions:

##### Explanation for choice of comparators:

The PRECICE study would compare combination treatment of duloxetine and web-based self-guided CBT (with or without phone-based MI) to duloxetine monotherapy. Such comparison would allow us to address one critical question, “Is combination treatment, consisting of duloxetine and web-based CBT, more effective than duloxetine alone?” Duloxetine has been shown to improve musculoskeletal pain conditions including fibromyalgia, osteoarthritis, and chronic low back pain.^28,29^ Similarly, CBT is effective in the management of similar musculoskeletal conditions.^33^ There are no currently published studies comparing combined duloxetine and web-based CBT against duloxetine therapy alone for CMP.

In addition, this study will also examine the use of phone-based MI to encourage completion of the eight learning modules within the web-based CBT program and to practice the newly learned pain coping skills in their daily lives. We hypothesize that phone-based MI will enhance treatment effectiveness.

##### Intervention description:

###### Duloxetine:

Prior to randomization, all participants will receive duloxetine 30 mg once daily and will return to the research clinic one week later to assess medication side effects and willingness to proceed with the study. Only those participants who are tolerating the 30 mg duloxetine and willing to go up on the dose to 60 mg once daily will be randomized to one of the three treatment arms. Regardless of treatment arms, all participants will take 60 mg once daily for 24 weeks. At week 13 and week 25, participants will return to the research clinic for outcome assessment.

To assure safety of study participants, the research assistant, who is not involved in data collection and randomization, will complete a symptom monitoring form at week 1, 2, 4, 13, and 25. The assistant will report every adverse effect to the study Medical Safety Officer. For adverse effects that require medical attention, the study Medical Safety Officer will directly contact the participant to provide immediate care and guidance.

At the last study visit (week 25), all participants will be provided duloxetine 30 mg once daily for 7 days and then discontinue the medication. Those who would like to continue duloxetine will be instructed to talk to their primary care provider (PCP). They will be given a 2-week prescription of duloxetine that is enough until they get a refill from their PCP. In addition, the Medical Safety Officer will send a message to the PCP with a short summary of the research study and to consider continuation of duloxetine.

Adverse effects of duloxetine include sedation, nausea, headache, and dizziness which are typically transient in nature.^47^ For those who do not tolerate the side effects, the medication will be reduced or discontinued with an appropriate 7 day tapering regimen to prevent discontinuation syndrome.

If a participant becomes pregnant while in the study, the rare side effects of duloxetine for the fetus late in the third trimester include breathing difficulties, seizures, temperature instability, feeding difficulty, vomiting, low blood sugar, jitteriness, irritability, and tremor.^48^ For this reason, participants will be required to complete pregnancy tests at baseline and at week 13 to avoid unnecessary exposure to duloxetine. Subjects who become pregnant will be asked to discontinue duloxetine with an appropriate tapering regimen.

###### Web-based Cognitive Behavioral Therapy (web-based CBT):

The web-based CBT program (pain TRAINER) is an automated program (i.e., users learn skills with interactive, personalized training without any therapist contact) that includes 8, 35- to 45-minute training sessions (Table 1). Each session provides an educational rationale and training in cognitive or behavioral pain coping skills drawn from face-to-face CBT.^49,50^ The sessions and features are controlled by a programming component that applies an “expert systems” approach. In the form of computerized tailoring algorithm, the program pairs decision rules with a knowledge database to simulate the behavior and judgment of an expert – in this case, a highly trained therapist experienced in delivering face-to-face CBT. The decision rules customize (tailor) participants’ experience in the program based on their responses and progress through the program. In other words, our web-based CBT retains the therapeutic components and processes (e.g., knowledge, collaborative skills training, self-monitoring, reinforcement, motivational enhancement, and working alliance) underlying the benefits of the face-to-face interventions on which they are based.^49^

Participants will complete one session per week (on average) over 12 weeks; this timing offers flexibility in completing sessions (e.g., allowing for personal or medical events to delay completion of some sessions, which we have found users prefer). Session 1 starts with an overview of the CBT program and the intervention’s therapeutic rationale. This overview is followed by training in the first pain coping skill: progressive muscle relaxation. Sessions 2–7 teach, respectively, brief relaxation skills (i.e., “mini-practices”), activity-rest cycling, pleasant activity scheduling, cognitive restructuring (“coping thoughts”), pleasant imagery, and problem solving. Session 8 reviews each skill to consolidate learning and teaches strategies for long-term skill use. Between sessions, participants are asked to practice their newly learned skill and any skills they learned in past sessions. The program also includes a feature to enhance engagement and facilitate practice. This feature is a section of the program that participants access to self-monitor their progress by reviewing and changing practice goals, recording practices and “coping confidence” (self-efficacy for managing pain), viewing graphic summaries of progress over time, and managing automated practice reminders.

For safety assessments, a trained research assistant, who collects information on medication side effects, will ask about emotional distress related to the web-based pain coping skills training using a protocol that includes a referral to the Medical Safety Officer if any concerns are present.

###### Phone-delivered Motivational Interviewing (MI):

The primary purpose of phone-delivered MI is to enhance participant’s motivation to engage in web-based CBT and continued practice of pain coping skills. Subjects randomized to the combination treatment with phone-based MI will receive six phone calls from an MI trained interventionist at week 3, 6, 10, 14, 18 and 22 of the study timeline (Table 2). Telephone sessions may run for 20 minutes.

The interventionist has a bachelor’s degree in healthcare management and has five years of experience as a research coordinator and Spanish interpreter. Given her background, the interventionist underwent rigorous training in MI with one of the co-authors (S.K.) who is a member of Motivational Interviewing Network of Trainers (MINT). The initial MI training phase consisted of an initial three-hour workshop with focus on key concepts and brief exercises to practice skills. The interventionist also read the main MI text and observed video demonstrations of MI. Following the initial workshop, the interventionist had weekly one-hour training sessions with the MINT trainer (S.K.) for 3 months, with an emphasis on skills practice and role playing. In addition, the interventionist completed two recorded Motivational Interviewing Treatment Integrity (MITI) coded practice sessions and received verbal and written feedback from the independent MITI coder. Throughout the length of the study, the interventionist and the MINT trainer (S.K.) will meet twice monthly. Each meeting will involve discussion of select study calls, troubleshooting challenging situations, and practicing MI skill set.

Similar to a previously tested MI protocol to encourage exercise,^51^ the MI intervention has three phases. The first phase includes the first two calls which will focus on strategies that enhance motivation to practice newly learned pain coping skills. It involves eliciting from the patient statements (i.e. self-motivational statements) that support the following: a) The patient’s recognition of the full nature and extent of the problem, b) The patient’s concern about how he or she is currently managing the problem, c) The patient’s intention of changing in the direction of adaptive pain management, and d) The patient’s optimism that changes are possible.

The second phase is devoted to strategies that strengthen commitment to practice newly learned pain coping skills regularly and consistently. Specifically, the third call would include helping the patient develop a plan for change (i.e., shift from why the patient should consider change to how the patient will make changes), communicating free choice and reviewing consequences of adaptive vs. maladaptive pain-related behaviors. The fourth phone call would involve asking for a commitment to practice new skills and a plan worksheet.

The third phase involving the last two calls are for follow-through strategies to prevent relapse. To review the changes that have occurred since the last session, the MI-trained interventionist will praise and reinforce all approximations of progress. She will also review behavioral indicators of motivation, patient’s responses to questions concerning reasons for making or maintaining changes, and barriers to adherence. She will again obtain a commitment to follow through on the new plan.

To assess treatment fidelity, 10% of all audiotaped MI sessions will be reviewed using the MITI 4.2^52^ by an independent MITI coder who is a member of MINT. The MITI is the most used tool for evaluating fidelity to MI.

###### Other treatments:

Participants may use the medications that they were on at study entry including opioid and non-opioid analgesics for pain control, including over-the-counter medications, prescribed medications, and dietary supplements. Medication usage will be assessed at each study visit to adjust for co-intervention differences between groups in the analyses.

##### Criteria for discontinuing or modifying allocated interventions:

Participants are free to withdraw from participation in the study at any time upon request. A participant may be discontinued from the study for significant study intervention noncompliance, lost to follow up or inability to contact the subject. The reason for discontinuation or withdrawal from the study will be recorded.

Adverse events associated with the study interventions are expected to be minor for the participants. For participants who become pregnant during the study, duloxetine will be discontinued with an appropriate tapering regimen to avoid rare side effects for the fetus. Subjects will be allowed to continue with the study. When a subject discontinues from the study intervention but not from the study, remaining study procedures will be completed as indicated by the study protocol.

If a clinically significant finding is identified after enrollment, the investigator and the Medical Safety Officer will determine if any change in participant management is needed. Any new clinically relevant finding will be reported as an adverse event (AE) or serious adverse event (SAE). At the time of study intervention discontinuation, data will be collected including the reason(s) for discontinuing the participant from the intervention and methods for determining the need to discontinue. If the participant is due to complete assessments within 2 weeks of being discontinued from the study intervention, those assessments will be administered at the time of discontinuation; if the next scheduled assessments are more than 2 weeks from the discontinuation date, the discontinued participant will wait for the next scheduled assessment. Thereafter, the participant will be included in all future scheduled assessments, even though not participating in the intervention.

##### Lost to follow up:

A participant will be considered lost to follow-up if he or she fails to return for one study visit and the study staff are unable to contact the participant after at least three attempts. If a participant fails to return to the clinic for a required study visit, the staff will attempt to contact the participant, reschedule the missed visit, counsel the participant on the importance of maintaining the assigned visit schedule and ascertain if the participant wishes to and/or should continue in the study. If necessary, study staff will utilize a participant provided alternate contact list to reach the participant. Before a participant is deemed lost to follow-up, the investigator or designee will make every effort to regain contact with the participant (where possible, three telephone calls and, if necessary, a certified letter to the participant’s last known mailing address or local equivalent methods). These contact attempts will be documented in the participant’s medical record or study file. Multiple methods including text messaging and/or email will be used to contact participants who drop out. If the participant continues to be unreachable, he or she will be considered to have withdrawn from the study with the primary reason of lost to follow up.

##### Outcomes:

Table 3 provides descriptions on primary, secondary, tertiary, and exploratory outcomes of the study.

###### Primary outcome:

The primary outcome measure of the study is the Brief Pain Inventory (BPI)-Global Pain Severity (GPS) score collected at weeks 13 and 25. BPI GPS is a self-report measure of pain severity and interference with proven reliability and validity across different pain conditions.^45,46^ It is defined as the average of BPI pain severity and BPI pain interference. BPI pain severity is the average of four items asking about current pain and worst, least, and average pain in the past week. BPI pain interference is the average of 7 items that rate how pain interferes with various activities.^53^ A higher score on the BPI pain interference indicates greater interference.

###### Secondary outcome measurements:

The secondary outcome measures will be assessment of BPI pain severity and BPI pain interference. Given that the BPI GPS is a composite of both BPI pain intensity and interference, it is possible that a beneficial effect or lack of effect on one component or the other may be missed. Evidence shows that composite endpoints permit only global, not component-specific, conclusion and are subject to misinterpretation.^54^ The treatment effects may be qualitatively different for different components of the composite.^54,55^ By analyzing each component of the composite, we are more likely to detect individual effects.

###### Tertiary and exploratory outcome measurements:

The tertiary endpoints will include the Global Rating of Change (GRC), Patient-Reported Outcomes Measurement Information System (PROMIS) pain intensity, PROMIS pain interference, and the Chronic Pain Coping Inventory (CPCI). The GRC assesses overall clinical response. It is consistent with the Initiative on Methods, Measurement, and Pain Assessment in Clinical Trials (IMMPACT) recommendations for a 7-item patient global change scale.^53^ This scale is sensitive to treatment-related improvements as it is modified to detect finer gradations of improvement.^56^ PROMIS pain intensity and pain interference are self-reported measures on physical health (fatigue, pain intensity, pain interference, physical function, sleep disturbance, pain behavior, and sleep-related impairment) and social health (ability to participate in social roles and activities).^57,58^ PROMIS measures were developed and validated with state of the science methods to be psychometrically sound. The CPCI is designed to assess the use of coping strategies that are typically targeted for change in multidisciplinary pain treatment programs.^59^

The exploratory endpoints will include the Generalized Anxiety Disorder 7-item scale (GAD-7), Patient Health Questionnaire 8-Item Depression Scale (PHQ-8), pain catastrophizing scale (PCS), PROMIS Adult measures (as described above), Opioid Morphine Equivalent (OME), health care service utilization, PHQ Anxiety-Depression Scale (PHQ-ADS), and frequency of practicing pain coping skills. The GAD-7 is a validated screening and severity measure for the most common anxiety disorders in primary care (generalized anxiety disorder, panic disorder, social anxiety, and post-traumatic stress disorder).^60,61^ Higher scores on the GAD-7 represent more severe anxiety symptoms. Clinical anxiety is defined as GAD-7 score of ≥10, a cut point validated in previous studies.^60,61^ The PHQ-8 is a brief self-administered scale that assesses major depressive disorder core symptoms and allows a score based on the total number and severity of depressive symptoms noted over the previous two-week period. Its validity (including telephone mode of delivery), feasibility, and capacity to detect changes of depressive symptoms over time are well established.^62–65^ Clinical depression is defined as PHQ-8 score of ≥10, a cut point validated in prior studies.^64,66–68^ PCS is a 13-item scale that describes the catastrophic thoughts and feelings that people may have in response to pain. The psychometric properties of PCS are well established^69–71^ including sensitivity to change.^72,73^ The total score ranges 0 (no catastrophizing) to 52 (severe catastrophizing). OME is a measure of daily dose of opioid use. We will use self-reported opioid type,^74,75^ medical record-based dosage and self-reported daily frequency to calculate the OME, reported in milligrams per day. The OME is calculated by multiplying dosage by daily frequency by a conversion factor for each opioid based on opioid strength.^76^

We will also extract data on use of different allied health care services from date of enrollment until the one-year anniversary utilizing EMR. The specific health care utilization measures include the number of new referrals to other specialties or allied health services, number of visits to each specialty or allied health services, and number of orthopedic or musculoskeletal surgeries. The specialties and allied health services include Orthopedic Surgery, Spine Center, Neurosurgery, Pain Specialty, Physical Medicine/Rehabilitation, Rheumatology, Integrative Medicine, Psychiatry (for medication, counseling, or both), Physical Therapy, Occupational Therapy, or quartet system for PCP to make a Psychiatry/Psychology referral.

PHQ-ADS is a composite of PHQ-8 and GAD-7 scores. PHQ-ADS is a single measure for assessing psychological distress in clinical practice and research.^77^ This is especially salient given the frequent co-occurrence of depression and anxiety. PHQ-ADS cut points of 10, 20 and 30 were shown to represent mild, moderate, and severe levels of psychological distress, respectively. We are using cut point of ≥20 to represent moderate level of psychological distress.

During the outcome data collection at weeks 13 and 25, we will ask participants how many days they practiced pain coping skills in the past 2 weeks (maximum of 14).^50^ To assess co-intervention effect, a treatment survey will inquire about specific treatments the patient has received (opiates and other analgesics, psychotropic medications and use of complementary and integrative health modalities such as acupuncture) for pain since the last follow-up.^68^

##### Participant timeline:

Table 4 illustrates the participant timeline.

###### Sample size:

For our main outcome, we have two primary analyses. The first primary analysis is the comparative effectiveness of the two combination treatment groups (with and without phone-based MI) against the duloxetine only group. The sample size was calculated using a two-sided t test at the 2.5% level of significance. The 2.5% level was chosen to adjust for Bonferroni correction of testing two main hypotheses. The estimated standard deviation (SD) for BPI Global Pain Severity score is 2.2.^78^ Assuming a moderate correlation of 0.5 between baseline and follow-up measures, we estimate that the SD in an analysis of covariance (ANCOVA) will be approximately 1.9. To detect a difference of −1 in BPI global pain severity score between the combination groups and duloxetine only group, 75 evaluable participants are necessary per group to achieve 90% power.

The second primary analysis is to compare the effectiveness of combination treatment with phone-based MI vs. combination treatment without the phone-based MI. To detect a difference of −1 in BPI Global Pain Severity, 75 evaluable participants are required per group to achieve 83% power at the 2.5% level of significance with a two-sided t test. Overall, 280 participants total will be recruited for all three groups combined after adjusting for Bonferroni correction for two main hypotheses and assuming a 20% loss to follow up.

##### Recruitment:

Patients are initially identified through the EMR based on inclusion eligibility criteria. The recruited patients had a recent primary care clinic visit over the past 12 months and had received one of the relevant ICD10 codes. Potential participants are prescreened via telephone using the inclusion and exclusion criteria. Selected participants will be scheduled for an in-person visit where the informed consent process and baseline assessments are performed.

Participants who consent to participate in the study but are not assigned to the study intervention or entered in the study are deemed screen failures. This includes those who fall out during the run-in phase prior to randomization.

#### Assignment of interventions: allocation

Participants will be randomized using the Research Electronic Data Capture (REDCap) Randomization Module to one of the three treatment groups. REDCap will help implement a defined randomization model within the study project by allowing us to 1) Define all of the randomization parameters; and 2) Create and upload a custom randomization table (i.e., allocation list). The table will serve as a lookup table for deciding how to randomize subjects/records. The module will also monitor the overall allocation progress and assignment of randomized subjects. Randomization will be stratified by opioid use and the number of pain sites (£2 versus ³3 sites) as number of sites has been associated with worse outcomes.^79,80^ A research assistant who will not be involved in the outcome assessment data collection will conduct the randomization.

#### Assignment of interventions: blinding

Data analysts including the principal investigator (PI) and co-investigators will be blinded to group assignment throughout the study.

#### Data collection and management:

The data team, who is blinded of the treatment group assignment, has the primary responsibility of analyzing outcome data throughout the study. At each study visit, participants will enter their responses to the assessment questionnaires within a REDCap secure web platform. Individual participant’s data in REDCap will serve as the electronic case report forms (eCRFs).

Clinical data (including AEs, SAEs, and expected duloxetine medication adverse reactions data) will be entered into a REDCap secure web platform for building and managing online databases and surveys. REDCap is a 21 CFR Part 11-compliant data capture system available through the Wake Forest School of Medicine Clinical and Translational Science Institute. The data system includes password protection and internal quality checks, such as automatic range checks, to identify data that appear inconsistent, incomplete, or inaccurate. Clinical data will be entered directly from the source documents.

#### Confidentiality:

Efforts, such as coding research records, keeping research records secure, and allowing only authorized people to have access to research records, will be made to keep information safe. Data will be used only in aggregate and no identifying characteristics of individuals will be published or presented. Research identification numbers will be used to uniquely identify each individual. Safeguards will be established to ensure security and privacy of participants’ study records. Appropriate measures will be taken to prevent unauthorized use of study information. Research records will be kept in a locked room at the research office. The files matching participants’ names and demographic information with research ID numbers will be kept in a locked computer file (password protected). Only trained and delegated study personnel will have access to these files. After the study is completed, local data will be stored with other completed research studies in a secured storage vault. After IRB approval for data sharing is obtained, a de-identified dataset that is in accordance with the Health Insurance Portability and Accountability Act (HIPAA) and other state and federal right to privacy laws will be developed.

#### Statistical methods:

The primary outcome of the study is the BPI GPS score collected at weeks 13 and 25. All participants will be analyzed according to randomized treatment assignment regardless of adherence to the treatment protocols. The two primary hypotheses are (H1) Combination treatment with and without phone-based MI is more effective than duloxetine monotherapy in improving BPI GPS, and (H2) Combination treatment with phone-based MI is more effective than combination treatment without phone-based MI in improving BPI GPS.

Research has shown that the use of ANCOVA with baseline measure as a covariate is an optimal method in both design and analysis of trials with a continuous primary outcome. ANCOVA is superior in terms of efficiency, precision, and power compared to the use of change score as the primary outcome. Therefore, the primary analysis will be week 13 and 25 BPI scores as a primary outcome while adjusting for baseline measurement.

We will fit a linear mixed model (LMM) to account for the correlation among the repeated measures. The model will include indicator variables for intervention arms, visit, and the interaction term. Covariates will include the pre-randomization measure of BPI GPS and the two stratification factors of opioid use and number of pain sites. Average follow-up BPI GPS for the three intervention groups will beestimated using least square means. Two different contrasts will be constructed to test H1 and H2 separately, each at the significance level of 0.025.

Several sensitivity analyses will be conducted to evaluate the robustness of the results. We will use inverse probability weighting (IPW) to account for missing data. We will identify the baseline characteristics that may be associated with lost to follow-up and derive weights for use in IPW analysis. Additional sensitivity analysis will include indicators of new pain related medication taken and any use of complementary treatment.

Secondary outcomes will include the two individual components of the BPI GPS: BPI pain severity and BPI pain interference. We will use similar LMMs described for the primary outcome analysis.

A subgroup analysis will be conducted using LMMs to evaluate the interactions between the intervention arm (with and without phone-based MI vs monotherapy) and baseline characteristics. The subgroups include comorbid psychological distress, opioid use, and number of painful body sites. The interaction will be tested at the 0.10 level of significance. A significant interaction is indicative of a potential moderating effect of a baseline characteristic such as psychological distress for predicting the mean BPI GPS score under combination therapy.

The tertiary and exploratory outcomes will be analyzed using similar LMMs described for the primary outcome analysis.

#### Oversight and monitoring:

##### Safety assessment:

A Safety Monitoring Committee (SMC) will be formed to assure human subject safety and study integrity. A group of two clinical investigators (one with expertise in clinical trials and one with expertise in behavioral-based intervention) and one biostatistician (PhD) will be recruited. The PI and the study team will meet monthly to review all serious, unexpected, and on-site AEs and make recommendations for any changes in reporting, consent, or study activities. The SMC will meet twice a year and will be provided with a report containing safety data summaries, patient demographics and compliance data, recruitment, visit schedules, missed visits, outcomes, Medical Event Forms, and any other adverse events. Each member of the SMC will be given a detailed progress report at least 2 weeks before the meeting. The SMC will be able to request specific information and analyses from the research team for monitoring purposes at any time during the study. The SMC will make recommendations to the PI regarding continuation, termination, or other modifications to the study based on the observed adverse events of the treatment under study. The PI will inform the National Institute of Nursing Research (NINR) project officer of any recommendations from the SMC.

##### Data monitoring:

Data will be entered by study team members via a REDCap secure web-based application developed by the Wake Forest Data Management (WFDM) team. Web-based scripted data entry forms will be utilized to guide staff through the administration of screening instruments administered via phone. Once a subject passes screening and is formally enrolled in the study, study staff will use a custom data collection and randomization engine to collect demographic information, receive the randomization by stratum, and validate that inclusion and exclusion criteria have been appropriately assessed. Only the study investigators, research team, Institutional Review Boards, the Food Drug & Administration (FDA), and the National Institute of Health (NIH) will be granted access to study records and data.

Real time data validation will be utilized to ensure data quality at the time of entry. Data and form checks will be completed by the WFDM team. Missing data will be monitored daily and the project manager on the study team side will be informed of missing data daily via “red flag” reminders in the database. The PI will discuss the rate and type of missing data during the monthly teleconferences.

##### Adverse event reporting and harms:

As part of the safety monitoring system, those who report AEs to study staff at any time will be referred to the Medical Safety Officer, who will identify, record, and manage the events. The study staff will report any study-related AE and/or any unanticipated problems involving risks to the Medical Safety Officer. For AEs that require medical attention, the Medical Safety Officer will directly contact the subject. Safety related events will be reported within 10 days, as required by the SMC and the Institutional Review Board (IRB) that are responsible for study oversight.

##### Auditing:

Clinical site monitoring will be conducted to ensure that the rights and well-being of trial participants are protected, that the reported trial data are accurate, complete, and verifiable, and that the conduct of the trial is in compliance with the currently approved protocol. Monitoring will be performed by an experienced auditor who has served as an independent site visit team leader for multiple NIH audit and site visits. The auditor will site visit the study staff office to ensure that study procedures are understood and carried out correctly, and the program will provide a mechanism to encourage the effective and standardized delivery of recruitment efforts, intervention programs, and the collection of appropriate and valid data.

##### Protocol amendments:

Any amendments to the protocol will be reported to the IRB for review and approval before changes are implemented to the study.

##### Dissemination:

The results of this clinical trial will be shared through publications in peer-reviewed journals and by oral/poster presentations at national and international scientific meetings. Both positive and negative findings will be disclosed.

## Discussion

The PRECICE study will address a significant issue faced in primary care clinics: how to treat chronic musculoskeletal pain most effectively. The use of combination treatment with medication and CBT can optimize pain-related treatment outcomes at the primary care level where most pain is managed. Ideally, face-to-face CBT would be used in treatment of CMP given known beneficial effect.^49^ Unfortunately, patients often face significant barriers to attending face-to-face CBT sessions including limited access to trained providers, travel requirements, and financial difficulties.^35^ By including web-based CBT, our study addresses a paucity of information on the effect of this modality on CMP.

Combination treatment has been effective and is recommended to treat conditions including depression and fibromyalgia,^81,82^ and other nonpharmacological approaches such as exercise combined with medication successfully treat chronic pain.^83^ Combining medication and psychological approaches is recommended for treatment of depression, and it has been shown to reduce pain in patients with coexisting depression and musculoskeletal pain.^84^ Overall, the study of the combined treatment and its effects on CMP are limited in number. Our pilot study showed promising effectiveness of combining medication with phone-delivered CBT in the treatment of chronic musculoskeletal pain.^85^ The PRECICE study will establish a causal association between the treatment and improvement of CMP. Furthermore, this study is the first to use motivational interviewing by a non-psychologist to encourage continued application of pain coping skills to sustain treatment benefits. Using motivational guidance can aid patients in maintaining practice of pain coping skills gained from web-based CBT; thus, allowing for benefits of the treatment beyond the acute phase.

If proven to be effective, the effects of a combined treatment for CMP will impact the health care system and the US overall. Costs related to health care utilization and lost productivity may decrease.^86,87^ Access to effective treatment at the primary care level would decrease the need for specialty services to manage pain. As part of the study, we will collect measures on health care utilization to assess benefits. Furthermore, an internet-based behavioral program (if made widely available) and the availability of generic medication may improve treatment access even by patients from lower socioeconomic background. The use of an interventionist without a formal degree in psychology increases the likelihood that phone-based support is scalable in the future.

Establishing the effectiveness of medication with CBT to treat CMP would validate the biopsychosocial model of understanding chronic pain. The model emphasizes the complex interaction between biological factors (central sensitization), psychological factors (thoughts and mood), and social factors (interpersonal relationships) which plays a role in management of chronic pain.^88^ Duloxetine is effective in addressing biological factors by reducing central sensitization.^26–29^ CBT affects central sensitization^34^ and modifies the mood and thoughts of patients including pain catastrophizing^89,90^ and pain coping^91,92^ which have important clinical implications for management of chronic pain. Duloxetine may indirectly modify psychological factors; by decreasing pain severity, patients may be able to better engage with and learn CBT skills for pain management. Duloxetine and CBT have been effective in reducing symptoms of comorbid depression and anxiety influencing pain as well.^28,29^ The scarcity of studies assessing the effectiveness of combined treatments has limited our understanding of chronic pain.

In summary, the PRECICE trial will address the current need for more effective management of CMP by PCPs. It will answer if combination treatment with duloxetine and web-based CBT should be the standard approach for patients with persistent pain despite the use of non-steroidal anti-inflammatory drugs and consultation with other specialties (e.g., physical therapy). Secondarily, the study will determine if a phone-based support to promote use of pain coping skills is effective in enhancing treatment outcomes. Finally, the study will raise additional hypothesis if combination treatment is more effective or less effective in certain subgroups of patients (e.g., comorbid anxiety or depression, gender, and ethnic groups).

### Trial Status:

Protocol version 3 was approved on December 17, 2020. Recruitment began on February 24, 2021, and is expected to be completed in March 2024.

## Supplementary Material

Supplement 1

## Figures and Tables

**Figure 1.** Participant Flow

**Table 2.** Content summary of Motivational Interviewing phone calls

Phone Session	Week	Content
1	3	Self-motivational statements
2	6	Optimism about change and intention to change
3	10	Plan for change & communicating free choices
4	14	Commitment to complete web-based modules and apply pain coping skills
5	18	Reinforce any approximation of progress & review reasons for maintaining changes
6	22	Barriers to adherence and applying pain coping skills

## Data Availability

The datasets generated and analyzed during the study will be available in the NIH Data Repository. De-identified data obtained during the study will be made available beginning after publication of the main findings of the study. Investigators interested in obtaining de-identified study data will be instructed to send a blank CD to the primary investigator for copying. The study will be conducted in accordance with the NIH Public Access Policy. The project will comply with all NIH HEAL Initiative Data Sharing policies. The study will comply with the NIH Data Sharing Policy and Policy on the Dissemination of NIH Funded Clinical Trial Information and the Clinical Trials Registration and Results Information Submission rule. The trial was registered at ClinicalTrials.gov, and results information will be submitted to ClinicalTrials.gov.

## Abbreviations

AE: Adverse event
ANCOVA: Analysis of covariance
BPI: Brief Pain Inventory
CBT: Cognitive behavioral therapy
CMP: Chronic musculoskeletal pain
CNS: Central nervous system
CPCI: Chronic Pain Coping Inventory
EMR: Electronic medical record
eCRF: Electronic case report form
FDA: Food and Drug Administration
GAD-7: Generalized Anxiety Disorder 7-item scale
GP: Global Pain Severity
GRC: Global Rating of Change
HIPAA: Health Insurance Portability and Accountability Act
IMMPACT: Initiative on Methods, Measurement, and Pain Assessment in Clinical Trials
IPV: In person visit
IPW: Inverse probability weighting
IRB: Institutional Review Board
LMM: Linear mixed model
MI: Motivational interviewing
MINT: Motivational Interviewing Network of Trainers
MITI: Motivational Interviewing Treatment Integrity
NIH: National Institute of Health
NINR: National Institute of Nursing Research
OME: Opioid Morphine Equivalent
PCP: Primary care provider
PCS: Pain catastrophizing scale
PHQ-8: Patient Health Questionnaire 8-item Depression Scale
PHQ-ADS: PHQ Anxiety-Depression Scale
PI: Principal investigator
PRECICE: Pain Response Evaluation of a Combined Intervention to Cope Effectively
PROMIS: Patient-Reported Outcomes Measurement Information System
RCT: Randomized control trial
REDCap: Research Electronic Data Capture
SD: Standard deviation
SMC: Safety Monitoring Committee
SPIRIT: Standard Protocol Items: Recommendations for Interventional Trials
WF: Wake Forest
WFDM: Wake Forest Data Management
