# Supplementary material for: Duloxetine and Cognitive Behavioral Therapy with Phone-based Support for the Treatment of Chronic Musculoskeletal Pain: Study Protocol of the PRECICE Randomized Control Trial
Source: Res Sq. 2024 Apr 15:rs.3.rs-3924330. Preprint. [Version 1] doi: 10.21203/rs.3.rs-3924330/v1 (PMC11065058; doi:10.21203/rs.3.rs-3924330/v1)
Supplement: Supplement 1 [file NIHPPrs3924330v1-supplement-1.pdf]

Tables

Tables 1, 3, and 4 are available in the Supplementary Files section.

Supplementary Files

This is a list of supplementary files associated with this preprint. Click to download.

- AdditionalFile1.docx
- AdditionalFile3.pdf
- Tables.docx
